# Validation of a self‐report adherence measurement tool among a multinational cohort of children living with HIV in Kenya, South Africa and Thailand

**DOI:** 10.1002/jia2.25304

**Published:** 2019-05-31

**Authors:** Rachel C Vreeman, Michael L Scanlon, Wanzhu Tu, James E Slaven, Carole I McAteer, Stephen J Kerr, Torsak Bunupuradah, Sararut Chanthaburanum, Karl‐Günter Technau, Winstone M Nyandiko

**Affiliations:** ^1^ Department of Health Systems Design and Global Health Arnhold Institute for Global Health Icahn School of Medicine at Mount Sinai New York NY USA; ^2^ Academic Model Providing Access to Healthcare (AMPATH) Eldoret Kenya; ^3^ Department of Child Health and Paediatrics School of Medicine College of Health Sciences Moi University Eldoret Kenya; ^4^ John W. McCormack Graduate School of Policy and Global Studies University of Massachusetts Boston MA USA; ^5^ Department of Biostatistics Indiana University School of Medicine Indianapolis IN USA; ^6^ HIV‐NAT Thai Red Cross AIDS Research Centre Bangkok Thailand; ^7^ Research Affairs Faculty of Medicine Chulalongkorn University Bangkok Thailand; ^8^ The Kirby Institute UNSW Australia Sydney Australia; ^9^ Department of Paediatrics and Child Health University of the Witwatersrand Johannesburg South Africa

**Keywords:** HIV/AIDS, children, adherence, Kenya, Thailand, South Africa

## Abstract

**Introduction:**

There are few data on adherence and low‐cost measurement tools for children living with HIV. We collected prospective data on adherence to antiretroviral therapy (ART) among a multinational cohort of children to evaluate an adherence questionnaire.

**Methods:**

We enrolled 319 children ages 0 to 16 years on ART in Kenya (n = 110), South Africa (n = 109) or Thailand (n = 100). Children were followed up for six months of adherence monitoring between March 2015 and August 2016 using Medication Event Monitoring Systems (MEMS ^®^) with at least one viral load measure. At month 3 and 6, children or their caregivers were administered a 10‐item adherence questionnaire. Repeated measures analyses were used to compare responses on questionnaire items to external adherence criteria: MEMS ^®^ dichotomized adherence (≥90% of doses taken vs. <90%), 48‐hour MEMS ^®^ treatment interruptions and viral suppression (<1000 copies/mL). Items associated with outcomes (*p *< 0.10) were coefficient‐weighted to calculate a total adherence score, which was tested in multivariate regression against MEMS ^®^ and viral suppression outcomes. Odds ratios (OR) and 95% confidence intervals (95% CI) were calculated.

**Results:**

Mean child age was 11 years and 54% were female. Children from Thailand (median age 14 years) were significantly older compared to Kenya (10 years) and South Africa (10 years). Prevalence of viral suppression was 97% in Thailand, 81% in South Africa and 69% in Kenya, while the prevalence of MEMS ^®^ adherence ≥90% was 57% in Thailand, 58% in South Africa and 40% in Kenya. Across sites, child‐reported adherence using the questionnaire was significantly associated with dichotomized MEMS ^®^ adherence (OR 1.8, 95% CI 1.4 to 2.4), 48‐hour treatment interruptions (OR 0.41, 95% CI 0.3 to 0.6), and viral suppression (OR 3.4, 95% CI 1.7 to 6.7). We did find, however, that different cut‐points for the adherence score may be context‐specific. For example, MEMS ^®^ non‐adherent children in Kenya had a lower adherence score (0.98) compared to South Africa (1.77) or Thailand (1.58).

**Conclusions:**

We found suboptimal adherence to ART was common by multiple measures in this multi‐country cohort of children. The short‐form questionnaire demonstrated reasonable validity to screen for non‐adherence in these diverse settings.

## 1 Introduction

In 2017, there were an estimated 1.8 million children under the age of 15 living with HIV, with 180,000 children newly infected, only 52% on life‐saving antiretroviral treatment (ART), and the vast majority living in low‐ and middle‐income countries (LMIC) 1. For children on ART, sustained levels of high adherence to treatment are required to achieve good treatment outcomes and transition into adolescence and adulthood 2, 3, 4, 5. Non‐adherence, which is often defined as taking less than 90% of prescribed doses, is associated with increased odds of virologic failure, drug resistance, various morbidities and mortality 6, 7, 8.

Estimates of adherence to ART among children vary widely within and across settings, which may in part be explained by heterogeneous methods for adherence assessment 9, 10. A systematic review of paediatric adherence to ART in LMIC found that among 17 studies, adherence estimates ranged from 49% to 100% and used a combined total of seven different assessment methods, with the most common being child‐ or caregiver‐report 10. In one of the largest prospective cohort studies of paediatric adherence, a study of 3304 children on ART in care at 26 sites in East Africa found high rates of adherence using 7‐day child‐reported missed doses, 7‐day caregiver‐reported missed doses and clinician pill assessment; across sites, between 94% and 98% of children were found to have “good” adherence, defined as either not reporting any missed doses or their clinician reporting two or fewer missed doses over the past month 11.

In many settings and studies, the validity of self‐ and caregiver‐reported adherence has not been established and may overestimate adherence levels compared to other measures such as pill counts, pharmacy refill records and electronic dose monitoring 12, 13, 14, 15, 16. Qualitative work shows that children and caregivers may feel reluctant to admit non‐adherence to health providers that are shaped by a variety of complex socio‐economic barriers to adherence 17, 18. In addition to common challenges associated with taking daily, lifelong medication 19, the stigma that often accompanies HIV and its treatment may contribute negatively to adherent behaviours, including psychological distress and not taking medicines to prevent disclosure of status to others 20, 21. Adherence to ART among children and adolescents is consistently associated with a host of factors, including gender, disclosure, attitudes about treatment, treatment complexity, mental health (of the child and caregiver), family dynamics (e.g. orphan status), timely clinic attendance and environment (e.g. rural vs. urban) 10, 22, 23.

Consistent and accurate adherence monitoring is an essential component of comprehensive HIV care. Adherence monitoring is particularly important for children transitioning into adolescent and adult HIV care settings and taking more ownership over their own treatment management; adherence in children and adolescents often decreases as they get older and presents additional clinical and behavioural challenges for HIV treatment and care 24. Identifying these children with adherence issues requires accurate assessment methods, especially as data on effective interventions for children and adolescents in resource‐limited settings to improve adherence to ART are emerging 25, 26. The International Association of Physicians in AIDS Care recommends routine self‐reported adherence assessment for all patients on ART, noting that while other assessment methods such as electronic dose monitoring are more closely associated with virologic indicators there are a number of disadvantages for their use in routine clinical settings (e.g. they may be burdensome for patients or incompatible with adherence‐promotion tools such as pill boxes) 27.

Given current recommendations around routine self‐reported adherence monitoring and the lack of data on its validity for the vast majority of children living with HIV in LMIC, studies are urgently needed to identify valid self‐report assessment tools. The purpose of this study was to prospectively describe adherence to ART among children in three LMIC—Kenya, South Africa and Thailand—and to validate a short adherence questionnaire that could be used as a screening tool to identify children needing additional adherence counselling and support.

## 2 Methods

### 2.1 Study design

We conducted a prospective study of adherence to ART among a multinational cohort of children. We enrolled children and their caregivers for six months of adherence monitoring between March 2015 and August 2016. Children were HIV‐infected, on ART (first or second‐line), between the ages of 0 and 16 years, and in care at one of three sites in Kenya, South Africa and Thailand (see *Setting* below). The only inclusion criteria for caregivers was that they reported being knowledgeable and involved in the child's HIV care, including medication taking. Convenience sampling was used as the small number of paediatric patients at each site restricted a random sampling approach. Potential study participants who met inclusion criteria were referred to study staff by clinicians, nurses and other clinic staff, as well as self‐referred through flyers placed in the clinics.

All participants were given Medication Event Monitoring Systems^®^ (MEMS^®^, MWV/AARDEX Ltd., Switzerland) that have a microcircuit that records the time and date of bottle opening and are often considered a highly reliable adherence measure given its correlation with virologic outcomes 28, 29. Only one MEMS^®^ was issued per participant; if participants had more than one medication, preference was given to fixed‐dose combinations and twice‐daily medications to be kept in MEMS^®^ bottles. Research assistants instructed participants on the use and care of MEMS^®^ during the study as well as how the MEMS^®^ recorded each time the bottle was opened. Thus, participants were not “blinded” to the MEMS^®^. Research assistants downloaded and reviewed with the participant MEMS^®^ adherence data at routine patient visits. If participants reported to clinic with a broken or dysfunctional MEMS^®^, they were issued a new MEMS^®^ and incomplete or missing data were censored. Children had a viral load test during the third month of follow‐up as part of the study, but may have had additional viral load tests as part of their routine clinical care.

At baseline, month 3 and month 6, participants were administered a 10‐item adherence questionnaire at their routine clinical visits. One version of the questionnaire for caregivers is included in the questionnaires are available from the authors on request. There is also a version of the questionnaire for children; the content of the questions is the same but the wording of questions is changed. The child version of the questionnaire is available upon request. The questionnaire tested in this study was developed through a multi‐staged, mixed methods strategy in Kenya 10, 16, 30, 31, 32. We conducted a literature review, cognitive interviews, and focus group discussions with children, caregivers, and providers to identify potential adherence questionnaire items. These were compiled in a longer adherence questionnaire that included 48 items, which we then tested among a cohort of Kenyan children and their caregivers using a similar strategy to this study with MEMS^®^ adherence as external adherence criteria. The ten best‐performing items were compiled in a “short‐form” adherence questionnaire that could potentially be used as a routine adherence screening tool. In the current study, the shorter version of the questionnaire was administered in the language of choice of the participants by different personnel at each site according to site protocols; for example, in Kenya the questionnaire was administered by the child's care provider, in South Africa by an adherence counsellor, and in Thailand by a study nurse. The questionnaire was administered to the child, caregiver, or both, depending on who reported primary responsibility for the child's medication‐taking. Responses to questionnaire items were recorded on paper and entered into a REDCap database 33. Individual‐level demographic and clinical characteristics of participants were collected from existing patient clinical records. We attempted to collect disclosure status of study participants as this has been identified as an important mediator of adherence; however, in clinical records disclosure status was only available for a minority of participants (36%).

All caregivers (parents or guardians) of participating children were asked to provide informed consent. Children eight years and older were also asked to give his/her assent to participate. This study was approved by the Indiana University Institutional Review Board as the implementing organization, and by local IRBs in Kenya (Institutional Research and Ethics Committee, Moi University School of Medicine and Moi Teaching and Referral Hospital), South Africa (Human Research Ethics Committee, University of Witwatersrand) and Thailand (Institutional Review Board, Chulalongkorn University).

### 2.2 Setting

This study took place at three paediatric sites (one peri‐urban and two urban) that participate in the global International Epidemiology Databases to Evaluate AIDS (IeDEA) collaboration. In Kenya, participants were recruited from a peri‐urban clinic that is part of the Academic Model Providing Access to Healthcare (AMPATH), an HIV treatment programme that follows over 10,000 HIV‐infected and exposed children through a network of 58 clinics in western Kenya. The AMPATH Busia clinic, located in Busia, a busy border crossing town on the Kenya‐Uganda border, is attached to the Busia District Hospital and provides care for about 400 children living with HIV and on ART. In South Africa, participants were recruited from an urban site—the Empilweni clinic at the Rahima Moosa Mother and Child Hospital in Johannesburg. The clinic is run by the Empilweni Services and Research Unit at the Department of Paediatrics and Child Health of the University of Witwatersrand, and is one of the largest paediatric HIV clinics in South Africa following over 1600 children. In Thailand, participants were recruited from another urban site—the ACTG Clinical Research Site of the Thai Red Cross AIDS Research Centre in Bangkok. The site is supported by the HIV‐Netherlands Australia Thailand Research (HIV‐NAT) organization and cares for a cohort of 195 children and adolescents living with HIV and on ART. No sites reported any drug stockouts or disruptions during the study period.

### 2.3 Data analysis

A minimum sample size of 100 participants at each site was calculated based on our objective to validate up to 10 items (N/k) at each site, and also give additional power for confirmatory analyses. Adherence by MEMS^®^ was calculated at month 3 (covering baseline to month 3) and month 6 (covering month 3 to month 6) in three ways: mean MEMS^®^ adherence, dichotomized MEMS^®^ adherence (≥90% of doses taken on schedule or <90%) and MEMS^®^ treatment interruptions of >48 hours (yes or no), consistent with other studies 34, 35. Viral loads were dichotomized to assess the proportion of participants who were virally suppressed, defined as a viral load <1000 copies/mL.

For basic demographics, child and caregiver ages were given with means (standard deviations) and compared across sites using ANOVA models, with pairwise comparisons utilizing Bonferroni adjustment to control for Type I error rates. If data are skewed, results are given as medians (interquartile ranges, IQR) and were analysed with Kruskal‐Wallis non‐parametric tests, using a Bonferroni correction on pairwise comparisons. Categorical variables were analysed with Chi‐Square tests, with Fisher's Exact tests being used when cell counts were small.

Separate analyses of the child form and caregiver form of the adherence questionnaire were conducted. Bivariate models were used to evaluate the association of responses on each questionnaire item at month 3 and month 6 to three external criteria: dichotomized MEMS^®^ adherence, 48‐hour treatment interruptions, and viral suppression. Odds ratios (OR) and 95% confidence intervals (95%CI) were calculated. We then tested the validity of the adherence questionnaire in two ways. First, we calculated a total adherence questionnaire score using a simple summation of participants’ responses, where “1” indicated a response representing a “non‐adherent” behaviour (e.g. responding that they had missed taking a dose in the past seven days) and “0” representing an “adherent” behaviour. Negative binomial generalized estimating equation (GEE) models were used to test the association between the total adherence scores with the three external criteria (dichotomized MEMS^®^ adherence, 48‐hour treatment interruptions, and viral suppression). Second, we calculated a weighted adherence questionnaire score. Variables associated with external criteria in bivariate analysis at *p* < 0.10 were included in a multivariate model. Items significant in multivariate regression (*p* < 0.10) were coefficient weighted to compute a weighted adherence score, which were then used in the negative binomial generalized estimating equation models as mentioned earlier. Models were adjusted for site (i.e. country), sex and age. We assessed the sensitivity of the questionnaire in predicting dichotomous adherence outcomes (MEMS^®^ adherence, 48‐hour treatment interruptions and viral suppression). To do so, we used a dichotomous value for child and caregiver reported adherence, categorizing each with the summation score of 0 versus ≥1 on the adherence questionnaire. As data for MEMS^®^ adherence and treatment interruptions were collected at more than one time point, repeated measures analyses were used for both these variables, which is why GEE models were utilized. All analyses were performed using SAS v9.4 (SAS Institute, Cary, NC).

## 3 Results

### 3.1 Demographics

A total of 319 child‐caregiver dyads participated in the study (Kenya, n = 110; South Africa, n = 109; Thailand, n = 100). There were slightly more female child participants (54%) and median child age was 11 years (IQR 8, 14). Children from Thailand (median age = 14) were significantly older than children from Kenya (median age = 10) and South Africa (median age = 10) (*p* < 0.05). Caregivers from Thailand (mean age = 44.3) were also significantly older than caregivers from Kenya (mean age = 38.7) and South Africa (mean age = 34.4) (*p* < 0.0001). The vast majority of caregivers were female (89%), which ranged from 97% of caregivers being female in South Africa to 79% in Thailand (*p* = 0.0002). Overall retention in the study was high; of the 319 child‐caregiver dyads recruited at baseline, 293 completed all study assessments (92%), which did not differ significantly by site.

### 3.2 Adherence outcomes

Examining dichotomized MEMS^®^ adherence, 48% of children were “adherent” (i.e. ≥90% doses taken) between baseline and month 3 and 51% were adherent between month 3 and month 6 (Table 1). About 40% of children had at least one MEMS^®^ treatment interruption of >48 hours between baseline and month 3 and 35% had at least one between month 3 and month 6. Among children with viral load measures taken during month 3 (n = 296), 82% were virally suppressed. Child and caregiver age and sex were not significantly associated with MEMS^®^ adherence, treatment interruptions or viral suppression, except for younger caregiver age that was associated with viral suppression (*p* = 0.01). Orphan status was only collected at the Kenya site, where being an orphan was associated with lower odds of dichotomized MEMS^®^ adherence (OR 0.33, 95% CI 0.14 to 0.77) and greater odds of MEMS^®^ treatment interruptions of >48 hours (OR 1.56, 95% CI 1.02 to 2.38).

**Table 1:** Adherence outcomes overall and by site

Site	Time period	
	Baseline – month 3	Month 3 to 6
Median % of MEMS^®^ doses taken		
All sites	88	90
Kenya	85	84
South Africa	87	93
Thailand	92	93
% of children with MEMS^®^ adherence ≥90%		
All sites	48	51
Kenya	44	40
South Africa	49	58
Thailand	52	57
% of children with MEMS^®^ treatment interruptions		
All sites	40	35
Kenya	36	38
South Africa	46	39
Thailand	33	30
% of children with viral suppression		
All sites	n/a	82
Kenya	n/a	69
South Africa	n/a	81
Thailand	n/a	97

Adherence outcomes varied by site. Children in Thailand and South Africa had slightly higher median MEMS^®^ adherence and a higher proportion of children with MEMS^®^ adherence ≥90% compared to and Kenya, particularly during months 3 to 6 (Table 1). The incidence of treatment interruptions was similar across sites. While 97% of children were virally suppressed in Thailand, 81% in South Africa and 69% in Kenya were virally suppressed. Interestingly, in this cohort of children MEMS^®^ adherence was not highly correlated with viral suppression overall or by site (*p* > 0.05).

When “adherent” was defined as an adherence score of 0—that is, the child or caregiver indicated no adherence issues in the questionnaire—according to child report, 52% were adherent at baseline, 57% at month 3 and 60% at month 6 (Table 2). According to caregiver‐report, 50% of children were adherent at baseline, 53% at month 3 and 60% at month 6 (Table 3). These responses are consistent when compared to “adherent” as defined by the proportion of children who had MEMS^®^ adherence ≥90%; between months 0 to 3, 48% were adherent and between months 3 to 6, 51% were adherent. Examining questionnaire responses by site, children in Kenya generally reported fewer adherence issues in their responses compared to children in South Africa and Thailand (Table 2), while caregivers in Thailand reported fewer adherence issues in their responses compared to caregivers in Kenya or South Africa (Table 3).

**Table 2:** Child‐reported adherence

Site	Time point		
	Baseline	Month 3	Month 6
% with adherence score of 0			
All sites	52	57	60
Kenya	73	80	84
South Africa	34	43	41
Thailand	48	46	51
% with adherence score of 1			
All sites	18	19	15
Kenya	6	1	2
South Africa	25	23	23
Thailand	24	36	23
% with adherence score of ≥2			
All sites	30	24	25
Kenya	22	19	14
South Africa	41	35	37
Thailand	28	18	26

**Table 3:** Caregiver‐reported adherence

Site	Time point		
	Baseline	Month 3	Month 6
% with adherence score of 0			
All sites	50	53	60
Kenya	45	51	71
South Africa	34	52	51
Thailand	72	57	55
% with adherence score of 1			
All sites	11	12	15
Kenya	11	5	13
South Africa	17	16	20
Thailand	5	16	12
% with adherence score of ≥2			
All sites	39	35	26
Kenya	45	45	17
South Africa	49	32	29
Thailand	23	27	33

### 3.3 Validation of questionnaire

Using the simple summation adherence score, across sites, child report and caregiver report were significantly associated with dichotomized MEMS^®^ adherence and MEMS^®^ 48‐hour treatment interruptions, while neither was significantly associated with viral suppression (Table 4). By site, there was some variation. In Kenya, child report was only significantly associated with one external criterion—viral suppression. In South Africa and Thailand, child report was significantly associated with MEMS^®^ 48‐hour treatment interruptions, while in Thailand it was also significantly associated with dichotomized MEMS^®^ adherence. Caregiver report was significantly associated with dichotomized MEMS^®^ adherence and MEMS^®^ 48‐hour treatment interruptions in South Africa and Thailand but not in Kenya.

**Table 4:** Association of simple summation questionnaire scores with external criteria

	Odds ratios (95% CI)			
	Kenya	South Africa	Thailand	All sites
Dichotomized MEMS^®^ adherence ≥90%				
Child form total score	1.09 (0.92, 1.30)	1.07 (0.96, 1.19)	1.37 (1.22, 1.54)*	1.16 (1.08, 1.26)*
Caregiver form total score	1.03 (0.93, 1.14)	1.13 (1.01, 1.28)*	1.13 (1.02, 1.25)*	1.08 (1.01, 1.15)*
MEMS^®^ 48‐hour treatment interruption (yes)				
Child form total score	0.86 (0.69, 1.08)	0.78 (0.64, 0.96)*	0.83 (070, 0.98)*	0.81 (0.73, 0.91)*
Caregiver form total score	0.90 (0.77, 1.04)	0.78 (0.64, 0.96)*	0.82 (0.70, 0.96)*	0.86 (0.77, 0.95)*
Virally suppressed (yes)				
Child form total score	1.31 (1.03, 1.66)*	0.92 (0.67, 1.27)	1.34 (0.71, 2.54)	1.16 (0.97, 1.38)
Caregiver form total score	0.99 (0.82, 1.18)	1.09 (0.78, 1.52)	1.43 (0.74, 2.76)	1.03 (0.88, 1.20)

* *p* < 0.05.

To identify which questionnaire items had the most power in predicting external adherence outcomes, we used the weighted adherence score. Overall, child‐reported adherence was significantly associated with all three external criteria: dichotomized MEMS^®^ adherence, MEMS^®^ 48‐hour treatment interruptions and viral suppression, while caregiver report was not significantly associated with any of them (Table 5). The items that were weighted and included in the adherence score were mostly consistent across child versus caregiver report and across the different external criteria. These items included the child or caregiver indicating the child missed doses, took doses late or took extra doses in the past week or month and reporting that they had difficulty in general taking the medicines on time. In analyses using the weighted adherence scores by site, we did not find any significant relationships to external criteria, which may be related to the smaller sample sizes and inadequate statistical power in the models when analysed by individual clinic.

**Table 5:** Association of weighted questionnaire scores with external criteria

	All sites, odds ratios (95% CI)	Questionnaire items and their weights in regression models
Dichotomized MEMS^®^ adherence ≥90%		
Child form total score	1.85 (1.41, 2.42)*	Missed doses in past month, 0.57 Missed doses in past week, 0.53 Problems with taking on time, 0.50 Late doses in past week, 0.41
Caregiver form total score	1.08 (0.83, 1.41)	Extra doses in past week, 0.78 Missed doses in past month, 0.47 Missed doses in past week, 0.38
MEMS^®^ 48‐hour treatment interruption (yes)		
Child form total score	0.41 (0.27, 0.62)*	Missed doses in past week, 1.25 Problems with taking on time, 1.17
Caregiver form total score	0.73 (0.50, 1.06)	Missed doses in past week, 3.57
Virally suppressed (yes)		
Child form total score	3.39 (1.72, 6.71)*	Extra doses in past week, 1.84 Missed doses in past month, 1.50
Caregiver form total score	1.56 (0.95, 2.57)	Problems with taking on time, 0.93 Missed doses in past month, 0.91

* *p *< 0.05.

The sensitivity of the questionnaire varied significantly by site against the three external adherence criteria (dichotomized MEMS^®^ adherence, MEMS^®^ 48‐hour treatment interruptions and viral suppression). Overall, the sensitivity of child‐reported adherence varied from 0.56 to 0.62, while caregiver reported adherence varied from summation scores of 0.54 to 0.64. However, in Kenya we found much higher sensitivity levels for child reported adherence (0.84 to 0.85) compared to in South Africa (0.39 to 0.48) and in Thailand (0.46 to 0.54). The sensitivities of the caregiver form did not differ significantly by site like the child form did.

## 4 Discussion

This study contributes to the limited data on paediatric adherence to ART in LMIC. Using electronic dose monitoring for prospective adherence monitoring, we found poor adherence was common among this multinational cohort of children. Only about half of children were able to maintain adherence rates above the recommended threshold of 90% of doses taken, on average more than a third of children experienced at least one treatment interruption of greater than 48 hours every three months, and 82% were virally suppressed. Although these outcomes varied by site with children in Thailand generally having higher adherence and viral suppression levels, our findings are concerning for children who require lifelong treatment and often experience additional clinical challenges associated with perinatal infection 24, 36. While our cohort was younger, studies suggest adherence in adolescents and young adults is generally lower compared to children and adults 9, illustrating the importance of maintaining and supporting adherence as children transition into adolescence.

Studies on adherence outcomes among children living with HIV in LMIC are heterogeneous 10. Two studies using MEMS^®^ and caregiver‐reported adherence (either through a three‐day recall or a 30‐day visual analog scale) among children in South Africa and Uganda found median MEMS^®^ adherence of 87% and 96% respectively and caregiver‐reported adherence between 97% and 100% 34, 37. We found similar MEMS^®^ adherence rates to South Africa in our multinational cohort, although children in Thailand specifically had MEMS^®^ adherence closer to those in Uganda. However, our study revealed much lower levels of adherence by caregiver report using our adherence questionnaire, suggesting that our questionnaire may be more sensitive in detecting adherence issues compared to standard approaches using one or two questions related to missed doses. While several of the questions from the short‐form questionnaire in our study that showed the greatest predictive power were related to missed doses, we also found questions asking about late or extra doses and general questions about adherence problems performed well.

The finding that MEMS^®^ adherence and viral suppression was generally lower among children in Kenya and highest among children in Thailand, with children in South Africa somewhere in the middle, may reflect the different contexts across the three settings in which adherence behaviours are constructed. It should be noted that any adherence assessment tool that inquiries about individual and clinic‐level barriers to adherence, as our tool does, should be specific and tailored to local contexts. While children in Kenya were in care at a well‐established HIV treatment programme, they were recruited from a peri‐urban clinic and were more likely to live in rural areas and to travel further to their HIV clinic compared to children at the South Africa and Thailand sites. Moreover, children at the Thai site were older and had been on treatment for longer due to the earlier rollout of ART programmes in Thailand compared to Kenya and South Africa. Unfortunately, we did not collect data on whether children were on a first‐ or second‐line regimen. Treatment regimen factors, such as side effects and pill burden, are important factors of adherence behaviours and outcomes. While our adherence questionnaire does include questions on medication‐related barriers to adherence, a limitation of our study is that we could not specifically examine these barriers by treatment regimen.

Overall, adherence improved over the course of follow‐up. For example, the proportion of children who reported perfect adherence increased from 52% at baseline to 60% at month, while the percentage of children with MEMS^®^ adherence ≥90% increased from 48% in the first three months to 51% in the last three months of follow‐up. Other study outcomes such as median MEMS^®^ adherence, MEMS^®^ treatment interruptions, and caregiver reported adherence supported this pattern of improved adherence. In previous studies in Kenya using adherence questionnaires and MEMS^®^, we also found that adherence tends to increase during the course of follow‐up [16, 32, 38]. This could be explained by a social desirability bias given the fact that participants are aware that their adherence is being closely monitored. Moreover, it is possible that participants opened the MEMS^®^ bottles without actually ingesting medication, which represents a limitation to adherence monitoring using MEMS^®^. Alternatively or in addition to a social desirability bias, the study procedures themselves likely constitute an adherence intervention. The adherence questionnaire and detailed MEMS^®^ adherence data provide opportunities for providers and families to better understand adherence barriers and problems and thus to strategize approaches to address them. In either case, we do not believe that this would necessarily impact our study's findings about the validity of the adherence questionnaire.

Despite evidence from this study and others that adherence to ART is a major challenge, there are still few low‐cost and validated tools to use to identify children who have poor adherence and can be targeted for interventions or counselling services. Electronic dose monitoring remains impractical in many clinical settings and other strategies such as pill counts, pharmacy refill data and clinician assessment often overestimates true adherence levels 10, 27. While viral load monitoring is becoming more routine in many LMIC and can be useful for identifying non‐adherence, this strategy cannot determine whether viral non‐suppression is due to adherence (as well as what type of adherence issues), drug resistance or some combination. Available data suggest drug resistance, particularly for older and more treatment experienced children, is increasingly common in LMIC 39, 40, 41. The frequency of treatment interruptions in this cohort as measured by MEMS^®^ highlights the potential risk for developing drug resistance with particular non‐adherent behaviours. Developing a low‐cost adherence questionnaire that can be used as a screening tool to identify children early who have potential adherence difficulties should be a major priority in paediatric HIV research. This study provides preliminary evidence for the validity of a short, 10‐item adherence questionnaire that showed good predictive power in identifying children who had adherence rates below 90%, treatment interruptions and viral non‐suppression.

An interesting finding of this study was that, generally, the questionnaire performed better when it was administered to the child themselves versus their caregiver. This study included a wide age range of children, from 0 to 16 years, and so children and caregivers presumably assumed the entire spectrum of levels of responsibility for medication taking, from younger children being completely reliant on caregivers for medication taking to older children taking responsibility for their medication taking. Our findings illustrate the importance of asking children about their medication‐taking and adherence from an early age and not solely relying on caregiver reports, which may be inaccurate because caregivers simply do not know (e.g. they are not always around the child when they take their medicine) or feel more pressure to report high rates of adherence to providers 42.

There was also some variation in the performance of the questionnaires by site. The questionnaire had the highest sensitivity in predicting adherence outcomes in Kenya, where it was associated with viral suppression, while it had lower sensitivity in South Africa and Thailand but was associated with MEMS^®^ adherence. Interestingly, viral suppression and MEMS^®^ adherence were not highly correlated in this study. While both viral suppression and MEMS^®^ adherence are typically considered the gold standards of adherence measurement, particularly in research settings, they may respond to different clinical and adherence‐related issues that may vary by region. As noted above, if drug resistance is higher in a certain context viral suppression rates may be less reflective of actual medication taking behaviour. Our finding that the adherence questionnaire was associated with viral suppression in Kenya but MEMS^®^ adherence in Thailand and South Africa may point to the importance of further adapting instruments or individual instrument items to specific contexts and populations. Moreover, the questionnaire was administered by different types of health providers at each site which introduces the potential that the questionnaire's validity varies according to provider type. Further refinement of the questionnaire in different contexts may be warranted but our study provides preliminary evidence to inform the number and type of questions that could be used in routine clinical adherence screening.

## 5 Conclusion

Supporting children living with HIV in adhering to ART is a pressing global challenge. While fewer children are being born with HIV every year, there are still millions of children currently living with HIV who require high levels of adherence to survive and thrive into adolescence and adulthood. Regular and accurate adherence evaluation is critical to this support. This study provides evidence for a 10‐item questionnaire that may be further revised and improved in different contexts for routine adherence screening in a clinic setting.

## Competing interests

The authors of this manuscript have no competing interests to report.

## Authors’ contributions

R.C.V., S.J.K., K.G.T. and W.M.N. led the conception and design of the study. M.L.S., C.I.M., T.B. and S.C. led the collection and management of data. R.C.V., M.L.S., W.T. and J.E.S. led the analysis of data. R.C.V. and M.L.S. led to the writing of the first draft of the manuscript. All authors contributed to reviewing and finalizing the manuscript and have approved of this version.
